# Retracted: Changes of Multisectoral Collaboration and Service Delivery in Hypertension Prevention and Control before and after the 2009 New Healthcare Reform in China: An Interrupted Time-Series Study

**DOI:** 10.1155/2022/9892367

**Published:** 2022-11-23

**Authors:** Journal of Healthcare Engineering

*Journal of Healthcare Engineering* has retracted the article titled “Changes of Multisectoral Collaboration and Service Delivery in Hypertension Prevention and Control before and after the 2009 New Healthcare Reform in China: An Interrupted Time-Series Study” [1] due to concerns that the peer review process has been compromised.

Following an investigation conducted by the Hindawi Research Integrity team [2], significant concerns were identified with the peer reviewers assigned to this article; the investigation has concluded that the peer review process was compromised. We therefore can no longer trust the peer review process, and the article is being retracted with the agreement of the Chief Editor.

The authors do not agree to the retraction.
